# Atresia of ileocecal junction, ileocecal valve: Rare variants of bowel atresia

**DOI:** 10.4103/0971-9261.57706

**Published:** 2009

**Authors:** Punit Srivastava, A. N. Gangopadhyay, D. K. Gupta, S. P. Sharma, Vijay D. Upadhyaya, Vijayendra Kumar, Richa Jaiman

**Affiliations:** Department of Pediatric Surgery, IMS, BHU, Varanasi, India

**Keywords:** Atresia, cecum, ileum, ileocecal valve, intestines

## Abstract

Atresia of ileocecal junction and isolated atresia of ileocecal valve are rare types of intestinal atresia with very few reports in literature. We report two such cases. Radiology showed dilated ileal segment and distal micro colon in both the cases. At laparotomy there was atresia of ileocecal junction in the first case and isolated ileocaecal valve atresia with normal ileocecal junction in the other case. Both the babies were managed by ileocolic resection with an end to end anastomosis. The prognosis of ileocecal atresias is satisfactory.

## INTRODUCTION

Intestinal atresia involving the ileocecal region is an extremely rare malformation, and the presence or absence of the ileocecal valve influences its surgical management and outcome.[1] Although about one-third of intestinal atresias are localized in the distal ileum, atresia of the ileocecal junction [ICJ] and atresia of the ileocecal valve [ICV] are the rarest variety. To the best of our knowledge, only four such cases have been reported in English literature.[2–4] We discuss both types: atresia of ileocecal junction segment with absent appendix and isolated atresia of ileocecal valve in which there was intact ileocecal wall segment with normal appendix.

## CASE REPORTS

### Case 1

**Figure 1:**
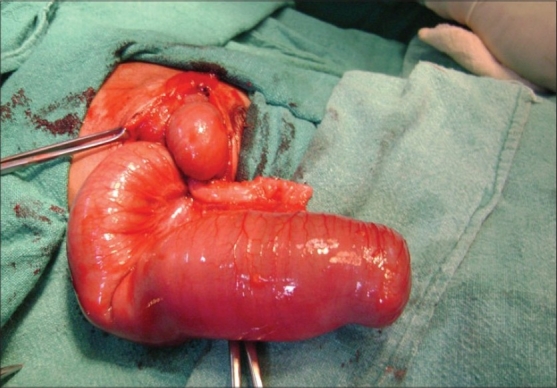
Intraoperative Picture: Absent Ileocecal Junction with Distended Ileum and Distal Microcolon

A one-day-old male neonate presented with bilious vomiting and abdominal distension since birth. The baby was born after a full term normal vaginal delivery, passed mucous per rectum and weighed 2.75 kg. Family history was non contributory; abdomen was distended and an X-ray of the abdomen revealed dilated bowel loops. Gastrograffin enema study showed microcolon. A diagnosis of distal small bowel atresia was made. At laparotomy, small bowel was distended with terminal ileocecal segment atresia, absent appendix and distal microcolon with V-shaped mesenteric defect [Figure 1]. The dilated part of ileal segment was excised and an end to end anastomosis was made. The post operative period was uneventful. He was discharged on 14th post operative day in a satisfactory condition. The baby was thriving on follow-up.

### Case 2

**Figure 2:**
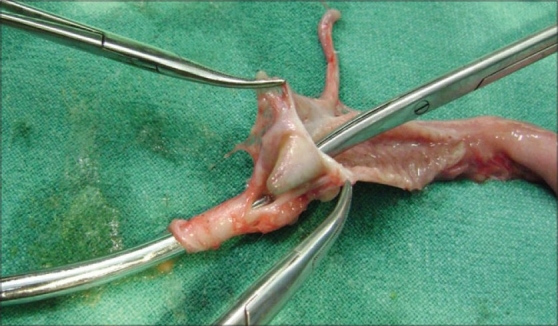
Resected Part of Ileocecal Segment: Atresia of Ileocecal Valve with Common Wall between Ileum and Cecum

A three-month-old baby presented with a prolapsing stoma in left iliac fossa, suggestive of sigmoid loop colostomy. He passed urine normally and mucous was discharged per rectum with adequate stoma function. He was earlier operated elsewhere and no previous operative records were available. The baby weighed 3.2 kg. Clinically, a atresia of recto-sigmoid region was suspected. Contrast enema showed contrast passing through normal recto sigmoid and colon and suggestive of micro colon. Contrast instilled into the stoma suggested dilated tapering loop towards right iliac fossa distally. There was diagnostic dilemma as the stoma in the left iliac fossa was loop ileostomy proximal to dilated atretic ileac segment, instead of sigmoid loop colostomy. At laparotomy, the ileum was dilated up to ileocecal junction with collapsed cecum without mesenteric defect. There was micro colon in continuity up to the terminal rectum. Saline injection to check the patency revealed block at the ileocecal junction. A diagnosis of isolated ileocecal valve atresia was made. Resection of ileocecal segment along with ileostomy loop and an end to end ileocolic anastomosis was made. Dissection of the specimen showed intraluminal obstructive wall at the ileocecal junction confirming ileocecal valve atresia[2,3] [Figure 2]. Pathological examination of the resected segments revealed complete absence of the ileocecal valve. The post operative period was uneventful. He was discharged home on 14th post operative day. The baby's condition was satisfactory at follow-up.

## DISCUSSION

Gaspard Bauhin (1560-1624), a botanist, anatomist, and physician, as well as Professor of Greek in Basle, Switzerland,[5] is presumed to be the first to describe the ileocecal valve. The ileocolonic junctional region exhibits many features considered characteristic of a gastrointestinal sphincter. The region generates a tonic pressure and exhibits responses to distension, nerve stimulation and pharmacological agents clearly different from adjacent ileum and colon. By preventing reflux of colonic contents the sphincter may serve to minimize colonization of the small bowel by large bowel bacterial flora.[6]

Atresia of the ileocecal junction is very rare with only four reported cases so far.[2–4] Ein *et al*,[2] and Cacciari *et al*,[3] reported three cases of atresia of the ileocecal valve in which there was a common wall between ileum and cecum which caused intraluminal obstruction. In these three infants the ileocecal segment and appendix were normal without any mesenteric defect. Our case number 2 was similar to this type. Ein *et al*,[2] resected the atretic segment and performed ileocolonic anastomosis without valve replacement as in our case. In contrast, Cacciari[3] resected only the middle part of the atretic ileocaecal valve, followed by ileocecal valve repair.

The fourth case, reported by Grassi *et al*,[4] was found in a 20-year-old, who had atresia of the ileocecal region and complete agenesis of the ileocecal valve. This patient was also treated by resection and Ileocolic anastomosis, with no valve reconstruction, followed by a good long-term outcome.

Clinical and experimental evidence suggest that ileal atresia could result from ischemic injury after the midgut has returned to the celomic cavity.[7] The V-shaped defect observed in the ileocecal mesentery of our case 1 which was suggestive of type 3a ileal atresia.[8] Both patients were well at follow-up after ileocolonic anastomosis. Preoperative gastrograffin enema in both cases demonstrated a microcolon, but there was no reflux of this contrast material into the terminal ileum. Ileocolic resection was carried out in both neonates. There is still a debate on ileocecal valve reconstruction in cases of valve atresia.
